# Characteristics and assembly mechanisms of tobacco-associated bacteria in typical tobacco-planting regions across China

**DOI:** 10.3389/fmicb.2026.1933656

**Published:** 2026-08-25

**Authors:** Hangkai Zhang, Zhenghan Lian, Yongjun Liu, Xiaozhan Qu, Yalong Xu, Jingjing Li, Yi Cao, Xueao Zheng, Yixiao Zhang, Peijian Cao, Liwei Zheng, Qiansi Chen

**Affiliations:** 1 China Tobacco Gene Research Center, Zhengzhou Tobacco Research Institute of CNTC, Zhengzhou, China; 2 Hunan Tobacco Science Institute, Changsha, China; 3 Tobacco Fujian Industrial Co., Ltd, Xiamen, China; 4 Guizhou Academy of Tobacco Science, Guiyang, China; 5 School of Agriculture and Biomanufacturing, Zhengzhou University, Zhengzhou, China

**Keywords:** biogeography, community assembly, life-history strategy, niche differentiation, tobacco

## Abstract

**Introduction:**

Plant-associated microbiota critically modulates host growth and environmental adaptation, yet assembly mechanisms, niche differentiation, and ecological strategies of bacterial communities inhabiting tobacco microhabitats remain poorly elucidated across geographical gradients.

**Methods:**

Here, we systematically characterized bacterial microbiome assembly across five tobacco-associated niches (bulk soil, rhizosphere soil, root, stem, and leaf) from seven typical tobacco-planting regions using 16S rRNA amplicon sequencing, genome annotation, and niche breadth analysis. The independent and interactive effects of geographical location and host compartment on community structure, and further compared genomic traits, functional profiles, and life-history strategies between specialist and generalist bacterial populations were quantified.

**Results:**

The results revealed a deterministic soil–plant continuum stratification of bacterial communities and diversity, with progressively simplified communities and decreasing alpha diversity from bulk soil to above-ground tissues, accompanied by progressive dominance of *Proteobacteria*. Geographical factors predominantly structured soil microbial communities via divergent edaphic properties, while host filtering acted as a universal dominant driver shaping endophytic microbiome assembly. Niche differentiation analysis demonstrated that niche-specialized bacterial ASVs overwhelmingly dominated all microhabitats and geographical sites, whereas generalist taxa only constituted auxiliary populations. Although specialist and generalist microbes exhibited highly conserved core genomic architectures and overall functional repertoires, they displayed distinct niche-specific functional divergence in metabolic pathways, stress resistance, and secondary metabolism across host compartments. Life-history strategy analysis further revealed that Y-strategist represented the core adaptive bacterial population, especially enriched in above-ground tobacco tissues.

**Discussion:**

Our study establishes a hierarchical dual-filtering assembly model for tobacco microbiota, clarifies the ecological differentiation and functional adaptation of specialist and generalist bacteria, and provides fundamental insights into the assembly rules and adaptive mechanisms of crop-associated microbiomes for future microbial resource utilization and agricultural microbiome regulation.

## 1 Introduction

Plant-associated microorganisms are essential drivers of host growth, environmental adaptation, and secondary metabolite accumulation (Schmidt et al., 2014; Chen et al., 2022; Huntsman and Leslie, 2024; Zhao et al., 2024). Distinct microhabitats along soil–plant continuum generate clear niche differentiation, which selectively filters and assembles specific microbial consortia (Bulgarelli et al., 2012; Edwards et al., 2015; Peng et al., 2023; Wang et al., 2026). Bacteria actively participate in nutrient absorption, stress resistance, and metabolic regulation of host plants (Vandenbrink et al., 2014; Backer et al., 2018; Cheng et al., 2023; Molinos-Albert et al., 2025; Zhang et al., 2026). With rapid development of high-throughput sequencing, microbial community assembly has been extensively characterized, and spatial niche, geographical condition, and host tissue type have been universally recognized as primary determinants shaping plant microbiota (Peiffer et al., 2013; Cregger et al., 2018; Su et al., 2023).

Tobacco is an economically important cash crop worldwide. To date, most tobacco microbiology studies have predominantly focused on microbial succession, mildew prevention, and flavor transformation during post-harvest storage and aging period (Wu et al., 2022; Kljajić et al., 2023; Xun et al., 2024; Tian et al., 2026). In contrast, community assembly and ecological characteristics of tobacco microbiota during cultivation stage remain poorly understood. In tobacco, most previous studies have been limited to single soil compartments (merely rhizosphere soil) (Gao et al., 2016; Lai et al., 2026; Xu et al., 2026), lacking systematic comparison across continuous multi-niche habitats, including bulk soil (BS), rhizosphere soil (RS), root (RT), stem (ST), and leaf (LF). Furthermore, most available investigations are based on single-site sampling, ignoring the geographical heterogeneity of tobacco-growing regions. Importantly, existing tobacco microbial studies generally concentrate on community composition description, whereas ecological strategies, assembly mechanisms, and the distribution patterns of specialist and generalist bacterial taxa remain largely unexplored.

The current knowledge gaps hinder an in-depth understanding of tobacco–microbe interactions under field conditions. Specifically, relative contributions of geographical factors versus host tissue filtering to bacterial differentiation remain quantitatively unclear in tobacco. Moreover, niche preference and functional differentiation of bacterial communities among various tobacco tissues have not been systematically validated. Additionally, life-history trade-offs and survival strategies of tobacco-associated microbiota are still undefined. The ecological distribution and adaptive differences between specialist and generalist bacterial taxa across belowground and aboveground niches also require further clarification. In the present study, we collected five continuous niches (BS, RS, RT, ST, and LF) from seven typical tobacco-planting regions across China. High-throughput 16S rRNA amplicon sequencing was applied to characterize bacterial community structures. This study aimed to quantify the relative contributions of geographical distance and host tissue filtering to bacterial assembly, reveal niche preference and functional differentiation of bacterial communities across multiple tobacco habitats, clarify microbial survival strategies and construct a life-history strategy framework for tobacco-associated bacteria, and distinguish the distribution patterns and ecological discrepancies between specialist and generalist taxa. Collectively, this study systematically characterizes the assembly mechanisms of multi-niche tobacco bacterial microbiota, fills the research gap concerning cultivated tobacco microbiomes, and provides fundamental theoretical support for the exploration of beneficial microbial resources and microbial regulation in tobacco cultivation.

## 2 Materials and methods

### 2.1 Study site and sampling process

BS, RS, RT, ST and LF samples of tobacco were collected at the middle leaf mature stage from seven typical tobacco-growing regions across China: Yuxi (YX, 24.35° N, 102.55° E, Yunnan Province), Zunyi (ZY, 27.72° N, 106.93° E, Guizhou Province), Chenzhou (CZ, 25.77° N, 113.03° E, Hunan Province), Longyan (LY, 25.12° N, 117.02° E, Fujian Province), Xuchang (XC, 34.04° N, 113.85° E, Henan Province), Weifang (WF, 36.71° N, 119.10° E, Shandong Province) and Harbin (MD, 45.75° N, 126.63° E, Heilongjiang Province). BS and RS were sampled randomly using the 5-point sampling method. Loose soil particles 1–20 mm away from root surface were collected via gentle shaking and kneading with sterile gloves, then immediately snap-frozen as BS samples. A 0–1 mm layer of tightly bound soil was carefully brushed from root surface using a sterile brush, then collected in a sterilized 50 mL tube to represent RS. All samples were immediately snap-frozen in liquid nitrogen, transported to laboratory on dry ice, and stored at −80 °C until further processing. For the determination of soil physicochemical properties, rhizosphere and bulk soil samples surrounding each plant were pooled, transported to laboratory, and air-dried at room temperature. Six biological replicates were performed for each sample.

### 2.2 Soil physiochemical analyses

Soil pH was determined using a pH meter via potentiometric method at a soil-to-water ratio of 1:2.5 (w/v). Nitrate and ammonium nitrogen (NO_3__N and NH_4__N) were quantified according to the method in our previous study (Xu et al., 2024), and total nitrogen (TN) content was determined by titration. Available phosphorus (AP) and total phosphorus (TP) were analyzed by colorimetry (Yuan et al., 2022; Westerman, (n.d.)), and available potassium (AK) and total potassium (TK) were measured using flame photometry (Yuan et al., 2022; Westerman, (n.d.)); total carbon (TC) was assayed by dry combustion via an elemental analyzer (Yuan et al., 2022; Westerman, (n.d.)).

### 2.3 Soil DNA extraction, PCR amplification and amplicon sequence processing

BS, RS, RT, ST, and LF overall bacterial community are analyzed through 16S rRNA gene amplicon. Genomic DNA was extracted from 0.25 g of each soil sample using the Soil DNA Extraction Kit (Findrop, China), following the manufacturer’s instructions. Epiphytic and endophytic microorganisms were recovered simultaneously from mature leaves, stems, and roots of tobacco plants following a modified surface sterilization and tissue separation method under aseptic conditions. Briefly, healthy tissues were harvested at the mature stage, rinsed briefly with sterile water to remove loose debris, and blotted dry with sterile filter paper. For epiphyte collection, 2–5 g of each tissue (leaves, stems, roots) was transferred into a 50 mL sterile centrifuge tube containing 30 mL of sterile phosphate-buffered saline (PBS, pH 7.2) supplemented with 0.1% (v/v) Tween 80. Samples were vortexed for 1 min, sonicated at 40 kHz for 10 min, and shaken at 200 rpm for 15 min on an orbital shaker. The tissue debris was then removed, and the eluate was centrifuged at 6,000 × g for 10 min at 4 °C. The resulting pellet, representing the epiphytic microbiota, was resuspended in 1 mL sterile PBS, frozen in liquid nitrogen, and stored at −80 °C for subsequent DNA extraction. For endophyte isolation, the recovered tissues (after epiphyte removal) were subjected to strict surface sterilization: 75% (v/v) ethanol for 45 s, 2.5% (v/v) sodium hypochlorite (NaClO) with one drop of Tween 80 for 8 min, followed by 75% ethanol for 30 s, and finally rinsed five times with sterile water (1 min each rinse). Sterility verification was performed by plating 100 μL of the final rinse water onto Luria–Bertani (LB) agar plates; no bacterial colonies after 3 days of incubation at 28 °C indicated successful surface sterilization. The sterilized tissues were blotted dry, ground into fine powder in liquid nitrogen using a sterile mortar and pestle, and stored at −80 °C as endophytic samples.

The qualified DNA samples were stored at −80 °C for subsequent sequencing analysis. The V5-V7 region of bacterial 16S rRNA gene was amplified using the universal primer pair 799F (5′-AACMGGATTAGATACCCKG-3′) and 1193R (5′-ACGTCATCCCCACCTTCC-3′). Purified amplicons were subjected to library preparation using the NEBNext^®^ Ultra™ II DNA Library Prep Kit for Illumina^®^ (New England Biolabs, USA), following the manufacturer’s instructions. Library quality and concentration were assessed using a Qubit^®^ 2.0 Fluorometer (Thermo Fisher Scientific, USA) and an Agilent Bioanalyzer 5400 system (Agilent Technologies, USA). Qualified libraries were subsequently sequenced on an Illumina NovaSeq platform, following standard procedures provided by Beijing Novogene Biotechnology Co., Ltd. (Beijing, China).

The 16S rRNA gene sequencing data of epiphytic and endophytic bacteria from LF, ST, and RT were separately merged for each tissue type. The combined datasets, integrating both epiphytic and endophytic microbial communities, were defined as the total bacterial microbiome associated with tobacco LF, ST, and RT, respectively. After data filtering with fastp (V1.0.1) (Chen et al., 2018), paired-end raw reads were merged, followed by dereplication, chimera detection, and generation of ASVs using USEARCH (V11.0.667) (Edgar, 2010). Taxonomic annotation of the representative sequences from ASVs was performed using the SINTAX algorithm with a 60% confidence threshold against the SINTAX-formatted RDP database. Sequences assigned to chloroplasts and eukaryota were subsequently removed from the 16S rRNA gene dataset for downstream analyses.

**Figure 1:**
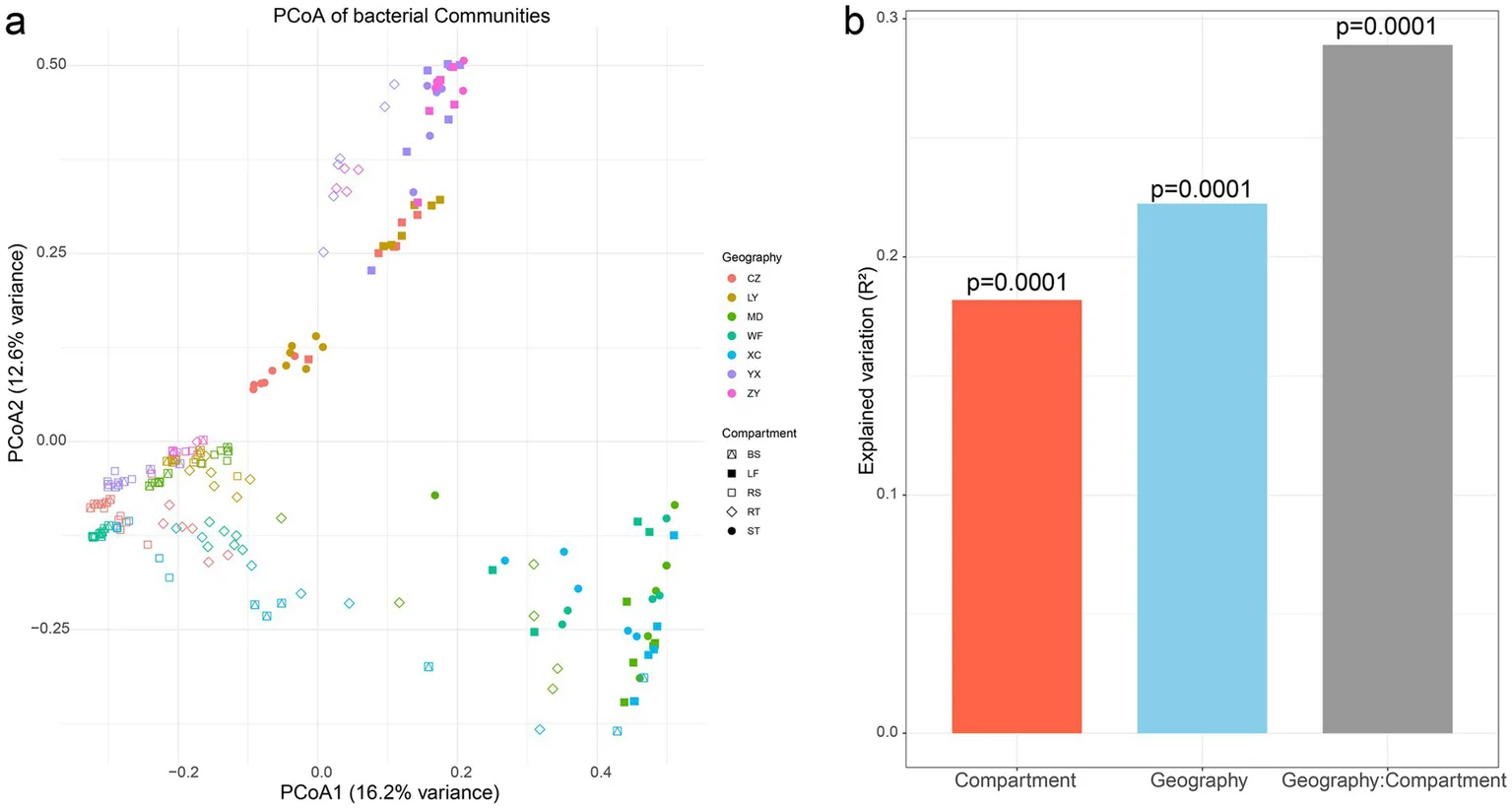
Beta diversity of bacterial communities and variance partitioning. **(a)** PCoA based on Bray–Curtis dissimilarity showing the bacterial community differentiation across geographical locations and plant-associated niches. The first two axes explained 16.2 and 12.6% of the total variation, respectively. **(b)** Sequential incremental *R*^2^ derived from PERMANOVA (adonis2, by = “terms”) showing bacterial community variation explained by geographic location (Geography), plant tissue compartment (Compartment), and their interaction. Terms were fitted sequentially in the order Geography → Compartment → Geography: Compartment interaction, generating non-overlapping additive explained fractions. All terms were statistically significant (*p* = 0.001, 999 permutations). The full model explained 69.31% of total community variation, with 30.69% residual unexplained variation (reported in the main text). Note that sequential Type I *R*^2^ values depend on the order of predictors in the model formula.

Rarefaction of the obtained abundance table to a sequencing depth of 5,000 reads (Supplementary Table S1). Beta diversity of bacteria communities was assessed based on Bray–Curtis, Jaccard, weighted and unweighted UniFrac distance matrices. PCoA was performed to visualize the dissimilarity of community composition among samples. Analysis of similarities (ANOSIM) and permutational multivariate analysis of variance (PERMANOVA/Adonis) were applied to test the significance of community structural differences between groups. Hierarchical clustering was conducted using the UPGMA algorithm to clarify the similarity of microbial community structures across samples. PERMANOVA with 999 permutations was performed on Bray–Curtis dissimilarities to quantify the relative contributions of geographic location (Geography), plant tissue compartment (Compartment), and their interaction to bacterial community variation. We applied sequential Type I sum-of-squares decomposition (by = “terms” in adonis2) to obtain non-overlapping incremental R^2^ values for each model term, which were visualized in Figure 1b. The full model explained 69.31% of total community variation, with the remaining 30.69% attributed to residual unexplained variation. Marginal Type III testing (by = “margin”) was also conducted as a supplementary analysis, yet this framework only provided significance for the Geography × Compartment interaction and could not separate independent marginal effects of the two main predictors, so it was not used for plotting. With the R package EcolUtils (v0.1, https://github.com/GuillemSalazar/EcolUtils), niche breadth indices were computed and compared against a null distribution derived from 1,000 random permutations. ASVs with observed niche breadth values above the upper 95% confidence interval (observed > uppCI) were classified as generalists, whereas those below the lower 95% confidence interval (observed < lowCI) were considered specialists (Gao et al., 2023; Yan et al., 2023).

### 2.4 Tobacco-associated bacterial genome search and annotation

Species-level genome was identified using representative partial 16S rRNA gene sequences derived from ASVs. These ASVs were aligned to 16S rRNA genes identified from GTDB database (release 214). Matches were determined via BLASTn, requiring ≥ 99% sequence identity and ≥ 90% coverage. In cases where a single ASV matched multiple genomes with the same similarity, the most complete and least contaminated genome was selected (Supplementary Table S2).

Open reading frames (ORFs) were predicted and translated using Prodigal (v2.6.3) (Hyatt et al., 2010). Functional annotation of each genome was then performed using MicrobeAnnotator (v.2.0.5) software (Ruiz-Perez et al., 2021), and then the KEGG Orthology (KO) numbers were mapped to KEGG metabolic pathways and manually curated. All proteins were queried against the curated KEGG Ortholog (KO) database using KofamScan (v1.3.0), with best matches were selected according to KofamScan’s adaptive score thresholds. Proteins without KO identifiers were extracted and further searched against other databases (e.g., Swissprot, curated RefSeq database or non-curated trEMBL database). KO identifiers for all proteins in each genome were extracted, and KEGG module completeness was calculated based on the total steps in each module, the required Kos for each step, and the KOs present in each genome. Results were aggrated into a comprehensive matrix summarizing module presence across all genomes (Supplementary Table S3). Based on previous studies (Malik et al., 2020; Cheng et al., 2024), screening criteria for the A, S, and Y life-history strategies were established. Analysis codes are available in Supplementary Tables S4–S7.

## 3 Results

### 3.1 Composition of tobacco-associated bacterial microbiota

The phylum-level taxonomic composition of bacterial communities was characterized across five tobacco-associated niches (BS, RS, RT, ST, and LF) at seven geographically distinct sites (YX, ZY, CZ, LY, XC, WF, and MD). Clear compartment-specific patterns and site-dependent variations in dominant phyla were observed along soil–plant continuum (Supplementary Figure S1). Across all samples, the bacterial communities were dominated by two core phyla: *Proteobacteria*, and *Actinobacteria*. These two phyla collectively accounted for >70% of the relative abundance in nearly all compartments. *Proteobacteria* was the most ubiquitous and abundant phylum, with its relative abundance increasing significantly from bulk soil to above-ground tissues. It constituted 40–60% of the community in bulk and rhizosphere soils, and became overwhelmingly dominant in ST and LF tissues, where it often accounted for >90% of sequences. *Actinobacteria* was a major component in bulk and rhizosphere soils (10–30%) and root tissues (10–40%), but its relative abundance declined sharply in ST and LF tissues, reflecting its adaptation to soil and rhizosphere environments. *Acidobacteria* was primarily found in bulk and rhizosphere soils (5–20%), consistent with its oligotrophic lifestyle (Fierer et al., 2007), and was nearly absent in above-ground plant tissues, indicating strong filtering by host.

The relative abundance of dominant phyla exhibited clear stratification along the soil–plant continuum, reflecting the increasing strength of host filtering. BS: The community was the most diverse, with *Proteobacteria*, *Actinobacteria*, and *Acidobacteria* as co-dominant phyla. Minor contributions from *Firmicutes*, *Gemmatimonadetes*, and *Verrucomicrobia* were also observed, reflecting the complex, oligotrophic soil environment. RS: Compared to BS, the rhizosphere showed a corresponding decrease in *Acidobacteria*. This shift reflects rhizosphere’s copiotrophic conditions, which favor fast-growing, carbon-responsive bacteria. RT: The root-associated microbiota community was dominated by *Actinobacteriota* and *Proteobacteria*, with the latter showing its highest relative abundance in this compartment. ST and LF: Above-ground tissues showed the most simplified community structures, dominated almost exclusively by *Proteobacteria*. The near-complete dominance of this phylum highlights the extreme environmental filtering imposed by stem and leaf phyllosphere, where only a narrow set of host-adapted lineages can persist. While the overall compartment-specific patterns were consistent across sites, significant site-dependent variations were observed in the relative abundance of dominant phyla, particularly in bulk and rhizosphere soils. Sites such as WFRS and LYRS showed unusually high abundances of *Firmicutes*, which may be related to local soil properties or environmental conditions. Some bulk soil samples (e.g., ZYBS, LYBS, WFBS, MDBS) exhibited higher proportions of *Acidobacteria*, likely reflecting differences in soil pH and nutrient availability across the sites. Despite these variations, the dominance of *Proteobacteria* in above-ground tissues was consistent across all seven sites.

### 3.2 Alpha diversity analysis of tobacco-associated bacterial communities

The alpha diversity of bacterial communities was assessed across five tobacco-associated niches (BS, RS, RT, ST, and LF) at seven geographically distinct sites, using Shannon and Simpson index (Supplementary Figures S2a,b). Across all sites, bacterial diversity exhibited a clear decreasing trend along the soil–plant continuum, with the highest diversity observed in bulk and rhizosphere soils, intermediate diversity in root tissues, and the lowest diversity in stem and leaf tissues. These compartments harbored the most diverse communities, with Shannon indices typically ranging from 3 to 7 in BS and RS. The Simpson indices were also consistently high (>0.8), reflecting high evenness in BS and RS. Diversity declined significantly compared to rhizosphere, with Shannon indices dropping to 2–5, and Simpson indices decreasing to 0.4–0.8 in RT, indicating the effect of host filtering. Above-ground tissues showed the lowest diversity, with Shannon indices often below 2 and Simpson indices as low as 0.3, consistent with extreme environmental filtering of the phyllosphere in ST and LF.

The magnitude of diversity decline varied markedly by compartment. Diversity in rhizosphere was generally comparable to bulk soil at most sites, indicating that rhizosphere environment supports a rich community. However, at some sites (e.g., LY), the rhizosphere showed slightly reduced diversity, suggesting site-specific variation in rhizosphere filtering. The most pronounced drop in diversity occurred between rhizosphere and root tissue, with Shannon indices decreasing by 1–3 units across most sites. Diversity in stem and leaf tissues was consistently low across all sites, with many samples showing near-monoculture dominance by a single taxon (reflected in Simpson indices close to 1). While the overall diversity gradient was consistent across sites, significant site-dependent variation was observed in magnitude of diversity within each compartment. Sites such as YX and ZY exhibited particularly high diversity in bulk and rhizosphere soils, with Shannon indices >6, suggesting favorable soil conditions. Despite these variations, the trend of decreasing diversity from soil to leaf tissue was universal across all seven sites.

### 3.3 Primary driving forces governing tobacco microbial community structure

To clarify whether geographical factors or host compartments act as the primary driver shaping tobacco microbial communities, we performed beta diversity and variation partitioning analyses to quantify their explanatory contributions. Principal coordinate analysis (PCoA) based on Bray–Curtis dissimilarity revealed that tobacco-associated bacterial communities were significantly structured by both geographical location and host compartment (Figure 1a). The first two axes of PCoA explained 16.2 and 12.6% of total variation in community composition, respectively. Clear separation of samples was observed along ordination axes, with communities clustering by geographical region and host compartment.

PERMANOVA revealed that compartment, geography, and their interaction each significantly structured bacterial community composition (all *p* = 0.001). Using a sequential testing framework with compartment fitted first, niche compartment alone explained 18.187% of community variation, geography explained an additional 22.23% after controlling for compartment, and their interaction explained a further 28.896%. The full model accounted for 69.313% of total community variation, with 30.686% remaining as unexplained residual variation (Figure 1b).

**Figure 2:**
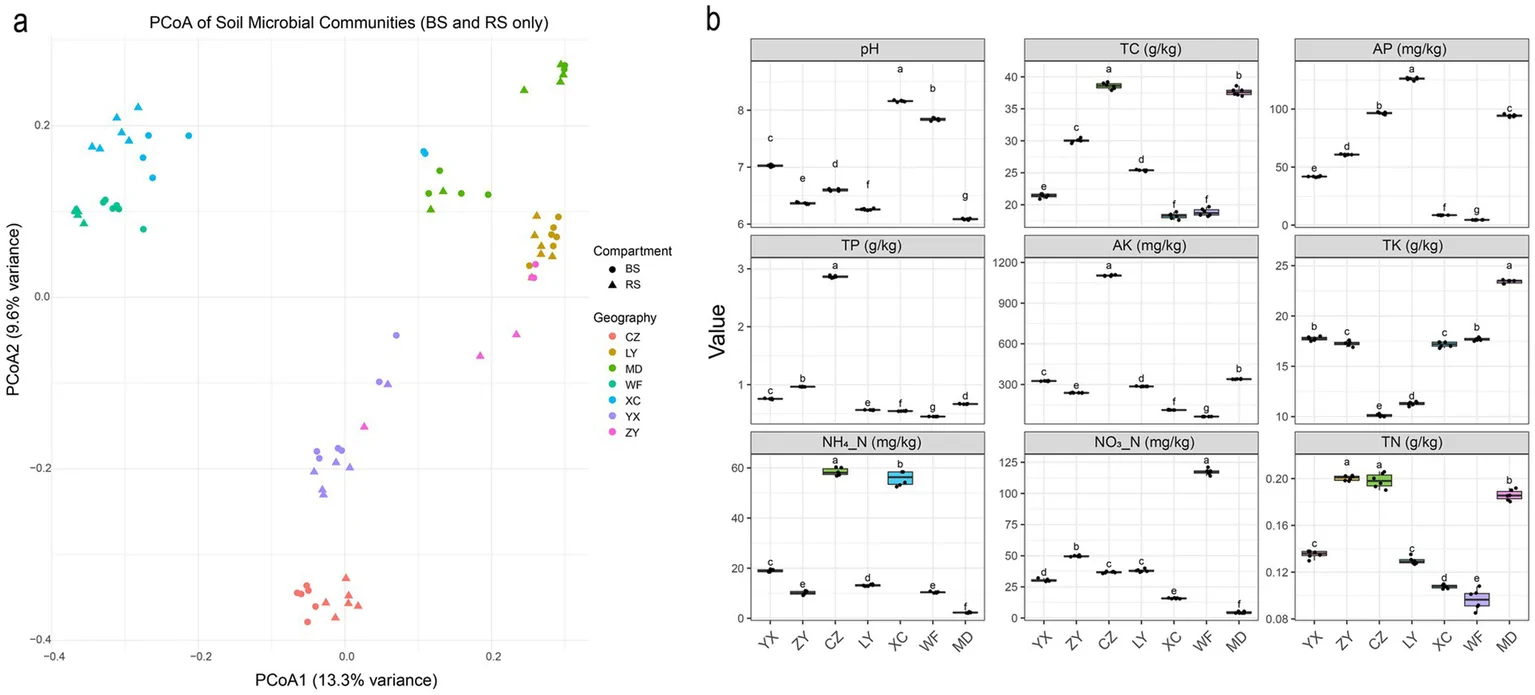
Soil bacterial community structure and physicochemical properties across seven geographical sites. **(a)** PCoA based on Bray–Curtis dissimilarity showing the differentiation of bacterial communities in bulk soil (BS) and rhizosphere soil (RS). The first two principal axes explained 13.3 and 9.6% of the total variance, respectively. **(b)** The variation of nine soil physicochemical variables (pH, TC, AP, TP, AK, TK, NH_4_-N, NO_3_-N, TN) among the seven sampling sites.

To further explore the drivers of tobacco-associated microbial community assembly, we focused on soil compartments (BS and RS), where environmental filtering is most pronounced. PCoA based on Bray–Curtis dissimilarity revealed a clear geographical clustering pattern of soil microbial communities (Figure 2a). The first two axes explained 13.3 and 9.6% of total variation, respectively, with samples primarily grouping by their geographical origin rather than by soil compartment (BS vs. RS). Consistent with this biogeographical pattern, physicochemical properties of soils, including pH, TC, AP, TP, AK, TK, NH₄-N, NO₃-N, and TN, exhibited distinct geographical gradients across seven sampling sites (Figure 2b).

### 3.4 Niche preference analysis of microbial species

**Figure 3:**
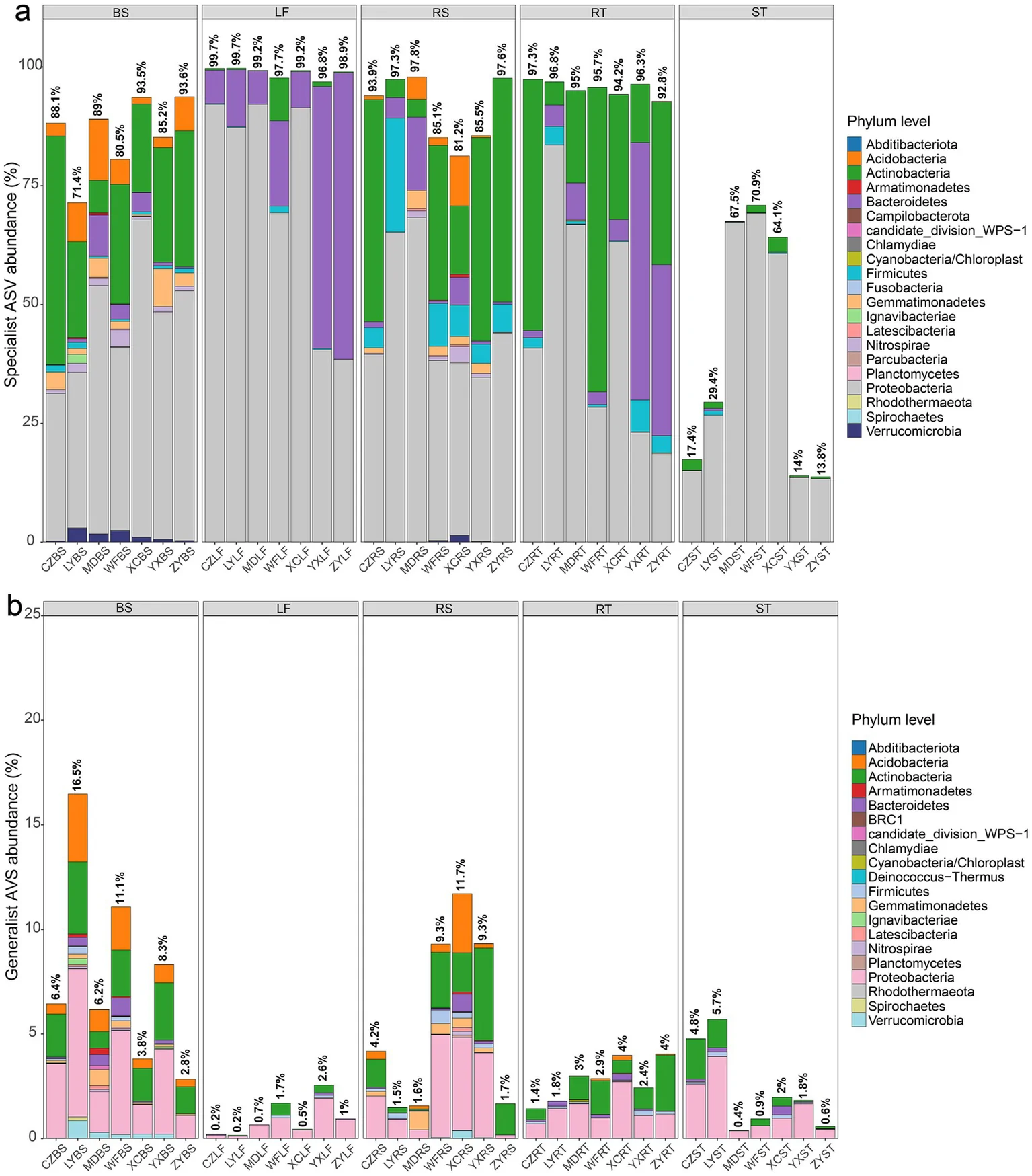
Phylum-level taxonomic composition of specialist and generalist bacterial ASVs across microhabitats and sampling sites. **(a,b)** The relative abundance of specialist and generalist ASVs at the phylum level in five tobacco-associated niches (BS, LF, RS, RT, ST) from seven geographical locations.

Niche breadth analysis was conducted to examine niche preferences of bacterial communities across distinct tobacco niches. (Supplementary Table S8) summarizes niche breadth metrics for co-occurring bacteria across BS, RS, RT, ST, and LF compartments of tobacco. At phylum taxonomic level, distinct abundance patterns were observed between specialist and generalist amplicon sequence variants (ASVs) across five tobacco niches (BS, RS, RT, ST, LF) and seven geographic sampling sites (CZ, LY, MD, WF, XC, YX, ZY) (Figure 3). Overall abundance disparity between specialist and generalist ASVs. Specialist ASVs exhibited markedly higher relative proportions than generalist ASVs in all tested niches and locations (Figures 3a,b).

The relative abundance of specialist phyla widely ranged from ~81.2% to nearly 100% across samples (Figure 3a), whereas generalist ASVs abundances remained consistently low, with most phylum-level generalist populations accounting for less than 20% and many taxa showing proportions below 5% (Figure 3b). This stark divergence indicates that host tissue-specific specialist microbial ASVs dominate the overall community, while generalist ASVs shared across multiple ecological niches constitute only a minor fraction of tobacco-associated microbiome. The most abundant specialist phyla included *Proteobacteria*, followed by *Actinobacteria*, *Firmicutes*, *Bacteroidetes*, *Acidobacteria*, *Planctomycetes*, *Verrucomicrobia*, and *Cyanobacteria*/*Chloroplast* (Figure 3a). Minor specialist phyla such as *Abditibacteriota*, *Armatimonadetes*, *Campilobacterota*, *Gemmatimonadetes*, *Ignavibacteriae*, *Latescibacteria*, *Nitrospirae*, *Parcubacteria*, *Rhodothermaeota*, *Spirochaetes*, and *candidate_division_WPS−1* were also detectable across samples but with substantially lower specialist ASV percentages (Figure 3a). Variations in the relative abundance of each specialist phylum existed among BS, RS, RT, ST tissues and different geographic sites, reflecting niche and geographic filtering effects on specialist microbial populations (Figure 3a).

Generalist ASVs were primarily affiliated with the same core phylum pool (*Proteobacteria*, *Actinobacteria*, *Firmicutes*, *Acidobacteria*, and *Bacteroidetes*) as specialist microbes, yet their relative abundances were drastically suppressed across all niches and locations (Figure 3b). Additional low-abundance generalist phyla comprised *Armatimonadetes*, *BRC1*, *Chlamydiae*, *Cyanobacteria*/*Chloroplast*, *Deinococcus−Thermus*, *Gemmatimonadetes*, *Ignavibacteriae*, *Latescibacteria*, *Nitrospira*, *Planctomycetes*, *Rhodothermaeota*, *Spirochaetes*, and *Verrucomicrobia* (Figure 3b). Only a small set of phyla (e.g., *Proteobacteria*) reached a generalist abundance of ~16.5% in individual samples; the vast majority of generalist phyla displayed proportions below 2%, with multiple taxa recorded at less than 1% across geographic and tissue gradients (Figure 3b).

### 3.5 Dissection of functional features in different host niches

**Figure 4:**
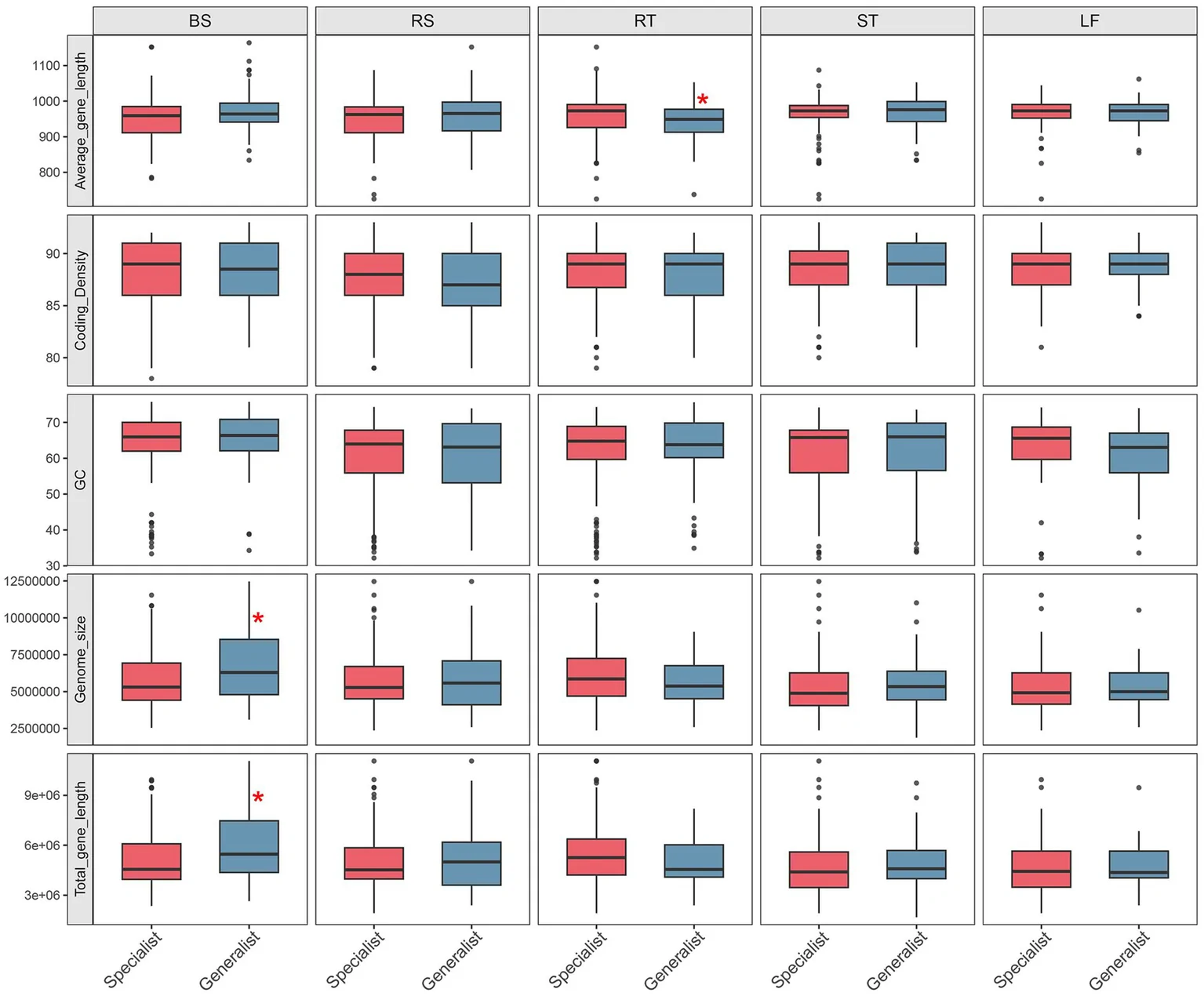
Comparison of genomic traits between specialist and generalist bacterial MAGs across five niches. Asterisks indicate significant differences between the two groups (*p* < 0.05). Niches include bulk soil (BS), rhizosphere soil (RS), root (RT), stem (ST), and leaf (LF). The Wilcoxon rank-sum test was adopted for statistical analyses.

Genomic structural traits were comparatively analyzed between specialist and generalist metagenome-assembled genome (MAG) across five niches (BS, RS, RT, ST, LF), including genome size, total gene length, average gene length, GC content and coding density (Figure 4). Overall, most genomic parameters showed no statistically significant differences between specialist and generalist strains in RS, ST and LF tissues. Significant intergroup discrepancies were only detected in two metrics at specific niches: (1) total gene length and genome size exhibited distinct differences between specialists and generalists in BS samples; (2) average gene length differed significantly between the two microbial groups only in RT niche. GC content and coding density remained comparable between specialist and generalist populations across all five niches without any significant variation. Collectively, the core genomic architecture of specialist and generalist microbes was largely conserved across most tobacco ecological niches, and only niche-specific selective pressure in BS and RT induced measurable divergence in partial gene and genome structural features.

**Figure 5:**
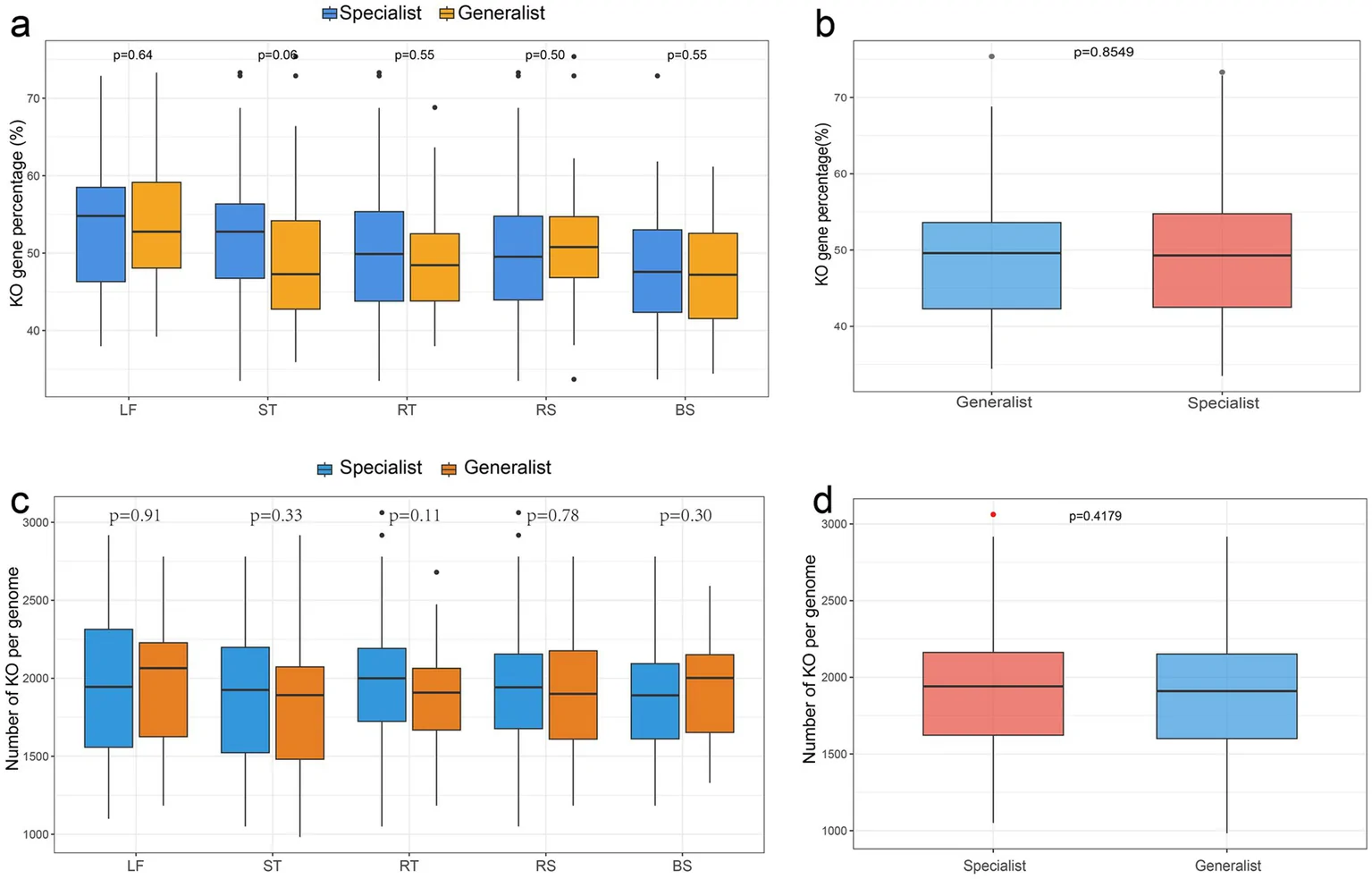
Comparison of KO functional gene profiles between specialist and generalist bacterial MAGs. **(a,b)** The percentage of KO genes in specialist (blue) and generalist (orange) genomes **(a)** presents comparisons within each of the five niches; **(b)** shows the overall comparison across all samples regardless of niche. **(c,d)** Total number of KO per genome between specialists and generalists **(c)** shows subgroup comparisons in individual niches and **(d)** displays global comparison across all MAGs. The Wilcoxon rank-sum test was adopted for statistical analyses.

We further compared proportion of KO genes and total number of KO carried by specialist and generalist microbes across five tobacco niches (BS, RS, RT, ST, LF) (Figure 5). Two sets of comparative analyses were performed: the relative percentage of KO genes (Figures 5a,b) and absolute count of KO per genome (Figures 5c,d). For KO gene percentage, Figure 5a presented comparisons between specialists and generalists in each niche, while Figure 5b aggregated all samples from five niches for integrated statistical analysis. Similarly, Figure 5c displayed the number of KO per genome separated by each niche, and Figure 5d combined data from all niches for overall comparison. Fisher test was applied to evaluate intergroup differences. All *p* values obtained from generalist and specialist comparisons ranged from 0.06 to 0.91, none of which reached significance threshold. Collectively, no statistically significant differences were detected between specialist and generalist populations in either relative proportion of KO genes or the total KO quantity at both individual niche and combined population levels.

PCoA based on Bray–Curtis dissimilarity matrices derived from the KO presence/absence profiles was performed to assess functional compositional divergence between specialist and generalist microbial populations. Two analytical strategies were implemented separately: (1) overall grouping without niche stratification (Supplementary Figure S3a, weighted KO matrix; Supplementary Figure S3c, unweighted KO matrix); (2) niche-stratified grouping for individual tobacco microhabitats (BS, RS, RT, ST, LF; Supplementary Figure S3b, weighted KO matrix; Supplementary Figure S3d, unweighted KO). Regardless of whether samples were analyzed as a unified pool or split by individual niches, specialist and generalist groups displayed partial overlap in the PCoA ordination space, without complete separation of the two microbial functional clusters.

Supplementary Figure S4 presents pairwise scatter plots illustrating detection rates of Fisher-test significant KO between specialist and generalist microbial populations across BS (Supplementary Figure S4a), RS (Supplementary Figure S4b), RT (Supplementary Figure S4c), ST (Supplementary Figure S4d), and LF (Supplementary Figure S4e). Functional category enrichment patterns of these niche-specific significant KOs varied markedly among distinct tobacco niches (Supplementary Table S9). For BS, significantly differentiated KOs were predominantly enriched in three core metabolic pathways: lipid metabolism, xenobiotics biodegradation, and energy metabolism. In RS, differential KOs showed prominent enrichment in amino acid metabolism, lipid metabolism, drug resistance, biosynthesis of other secondary metabolites, and metabolism of cofactors and vitamins as well as glycan metabolism. RT-specific significant KOs covered the broadest functional spectrum, including amino acid metabolism, pathogenicity, drug resistance, biosynthesis of terpenoids and polyketides, biosynthesis of other secondary metabolites, metabolism of cofactors and vitamins, and carbohydrate metabolism. ST-associated differential KOs were mainly concentrated in carbohydrate metabolism, glycan metabolism, and biosynthesis of terpenoids and polyketides. LF only displayed two enriched functional modules of divergent KOs, namely glycan metabolism and lipid metabolism.

### 3.6 Construction of life-history strategy framework for tobacco-associated microbiota

**Figure 6:**
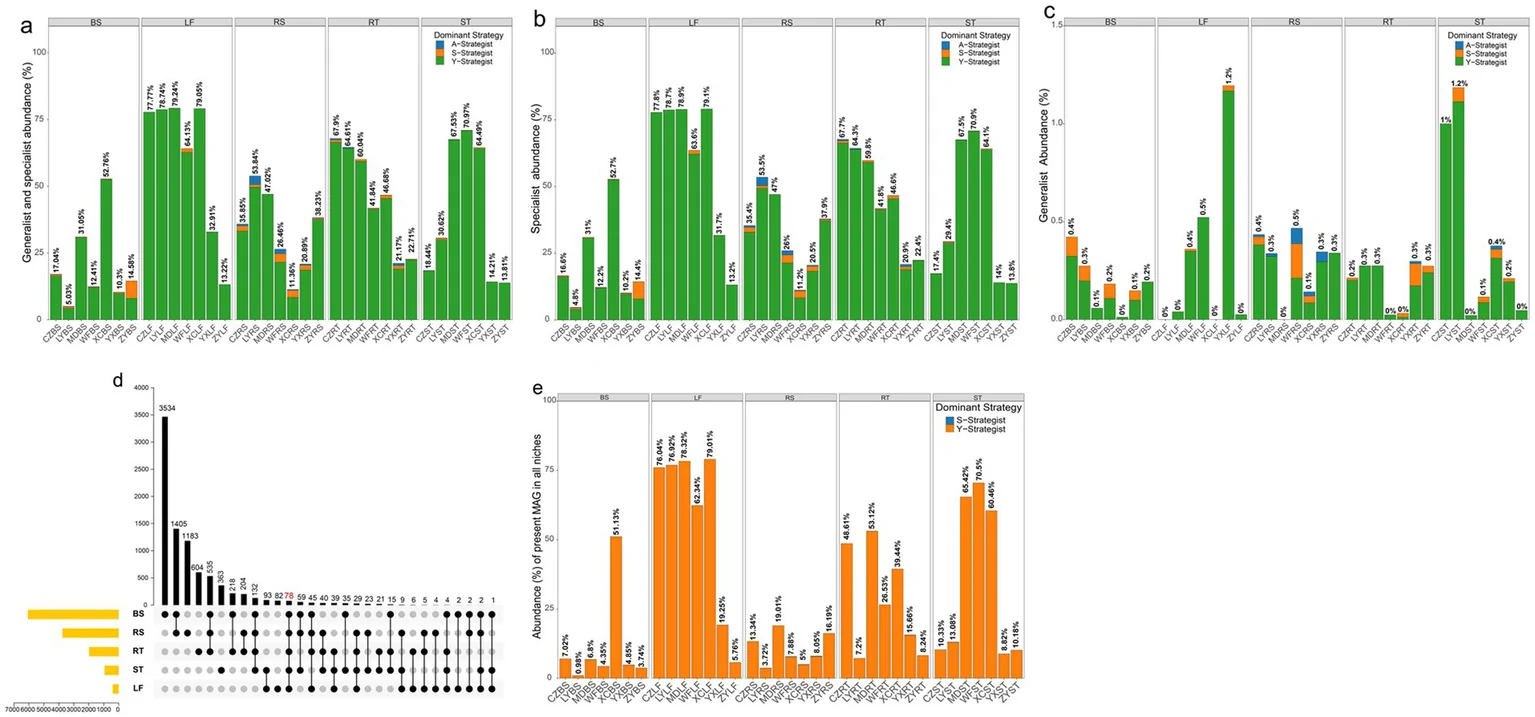
Relative abundance of A-, S-, and Y-strategists among specialist, generalist and core bacterial ASVs across niches and geographical sites. **(a)** The proportion of three life-history strategists in specialist and generalist ASVs. **(b)** Life-history strategy composition restricted to specialist ASVs only. **(c)** Life-history strategy composition restricted to generalist ASVs only. **(d)** Occupancy distribution of microbial MAGs across five tobacco-associated niches (BS, RS, RT, ST, LF). The number above each bar represents count of MAGs detected in a given number of niches. The 78 MAGs that occurred ubiquitously across all five microhabitats were defined as core microbiome members. Dots in the lower panel indicate the presence of each MAG in individual niches. **(e)** The relative abundance of core MAGs assigned to S- and Y-strategists across seven geographical sites and five microhabitats.

We further classified ASVs into generalist and specialist taxa, and subsequently assigned each taxon to three life-history strategies (A-strategist, S-strategist, and Y-strategist). Then the relative abundance of each strategist among total ASVs across five niches (BS, RS, RT, ST, and LF) and multiple geographical locations (Figures 6a–c) were calculated.

Across all niches and sampling sites, Y-strategists constituted the predominant component in both generalist and specialist microbial pools (Figure 6a). The proportion of Y-strategists was consistently higher in specialists than in generalists (Figures 6b,c). By contrast, A-strategists and S-strategists only occurred sporadically across niches, with extremely low relative abundances, and did not form dominant populations in any compartment. The relative proportions of generalists and specialists exhibited strong heterogeneity across geographical regions and host-associated microhabitats. Microhabitat exerted a clear filtering effect on these two ecological groups. Specifically, specialist populations maintained persistently high relative abundances in LF compartment across most locations, while the relative abundance of specialists was generally much lower in BS (Figure 6b). In addition, substantial variation in the abundance patterns of generalists and specialists was observed among different geographical sites within the same microhabitat (Figures 6b,c).

We first identified core microbial MAGs, which were defined as those consistently detected across all five host-associated niches (BS, RS, RT, ST, and LF). The occupancy analysis revealed a strongly skewed frequency distribution of MAGs across niches (Figure 6d). The vast majority of microbial MAGs were restricted to only a small number of niches, with 3,534 MAGs exclusively occurring in a single niche. Only a small subset of taxa was widespread across multiple compartments. Specifically, just 78 MAGs were present in all five microhabitats and were classified as core members of the microbiome.

We further quantified the relative abundance of these core MAGs in each sampling site and microhabitat (Figure 6e). The core MAGs were overwhelmingly dominated by Y-strategists, while S-strategists only contributed a negligible proportion of total core community across all samples. The relative abundance of core MAGs exhibited striking microhabitat differentiation. LF compartment harbored the highest proportion of core microbes, with most sampling sites recording core MAG abundances exceeding 60%, and several locations reaching nearly 80%. In contrast, BS contained the lowest relative abundance of core MAGs, with values mostly below 10%. Root-associated compartments (RS and RT) displayed intermediate levels of core MAG abundance, with substantial variation observed among different geographical sites within the same microhabitat.

## 4 Discussion

In the present study, we systematically characterized the assembly patterns, diversity gradients, driving factors, niche differentiation, genomic functional traits, and life-history strategies of tobacco-associated bacterial microbiomes across five continuous niches and seven geographical locations. Our multi-dimensional results reveal a hierarchical and deterministic assembly mechanism of plant microbiota, where host compartment filtering serves as the dominant universal driver governing microbiome structure, niche differentiation, and functional adaptation, while geographical environment acts as a secondary modulator that induces fine-scale community variation.

Consistent with the general paradigm of plant microbiome assembly, our results demonstrated distinct phylum-level community stratification along soil–plant continuum of tobacco (Supplementary Figure S1). *Proteobacteria* and *Actinobacteria* constituted the core bacterial taxa throughout all microhabitats (Supplementary Figure S1), consistent with their widespread dominance in terrestrial plant-associated microbial systems (Lundberg et al., 2012; Turner et al., 2013; Liu et al., 2025; Ruan et al., 2026). Notably, we observed a progressive community simplification from bulk soil to above-ground tissues, accompanied by a drastic shift in dominant microbial phyla (Supplementary Figure S2). Bulk and rhizosphere soils harbored diverse co-dominant bacterial groups including *Acidobacteria*, *Gemmatimonadetes*, and *Verrucomicrobia* (Supplementary Figure S1), reflecting the complex oligotrophic soil environment that sustains heterogeneous microbial populations. In contrast, above-ground stem and leaf tissues were overwhelmingly dominated by *Proteobacteria*, with nearly complete depletion of soil-adapted oligotrophic taxa such as *Acidobacteria* (Supplementary Figure S1). This compartment-dependent taxonomic stratification strongly indicates intensified host filtering pressure from below-ground to above-ground niches (Philippot et al., 2013; Edwards et al., 2015). Endophytic microhabitats, especially the phyllosphere, impose rigorous abiotic and biotic constraints including limited nutrient availability, strong oxidative stress, and host immune selection, thereby filtering out most soil-derived microbes and retaining only host-adapted specialist lineages (Jacoby et al., 2017). Such deterministic community turnover indicates that host microhabitat specificity is the fundamental prerequisite for shaping tobacco bacterial community composition.

Alpha diversity patterns further corroborated the hierarchical host filtering effect on tobacco microbiota (Supplementary Figure S2). Bacterial community diversity exhibited a universal declining gradient from bulk soil and rhizosphere soil to root, stem, and leaf tissues across all geographical sites (Supplementary Figure S2). The highly diverse and even microbial communities in soil compartments reflect the relatively open and nutrient-heterogeneous soil habitats supporting extensive microbial colonization. The sharp diversity reduction occurring at the soil–root interface represents the most critical screening stage of host selection (Supplementary Figure S2), whereas the consistently low diversity in stem and leaf tissues further demonstrates the extreme filtering effect of above-ground endophytic niches. Although geographical variation induced minor differences in microbial diversity among soil samples from different sites, the universal soil-to-leaf diversity decline across all locations verified that host compartment filtering overrides geographical environmental variation in structuring tobacco microbial alpha diversity patterns.

Beta diversity and variation partitioning analyses further clarified the relative contributions of host selection and geographical environment to microbial community assembly (Figures 1a,b). Both host compartment and geographical location significantly structured bacterial community dissimilarity, with their interactive effect explaining the largest proportion of community variation (Figure 1b), indicating that microbial assembly is co-modulated by host niche filtering and regional environmental adaptation. For soil microbial communities specifically, geographical divergence was more prominent than compartment differentiation, as bulk soil and rhizosphere soil samples clustered strongly by sampling sites rather than by microhabitat types (Figure 2a). This spatial biogeographical pattern is closely coupled with site-specific soil physicochemical properties, including pH, nutrient content, and ion availability, which shape unique local soil microbial pools (Figure 2b). Collectively, these findings support a dual filtering assembly model for tobacco microbiota: geographical environmental factors primarily determine the regional soil microbial species pool, while host compartment filtering further screens and reshapes microbial communities to form niche-specific endophytic microbiomes, as previously documented (Ikram et al., 2026).

Niche breadth analysis distinguishing specialist and generalist microbes provided novel ecological insights into tobacco microbiome assembly (Supplementary Table S1). Our study demonstrated that specialist ASVs overwhelmingly dominated the tobacco-associated bacterial community across all microhabitats and geographical sites, while generalist microbes only constituted a minor auxiliary component (Figures 3a,b). Soil compartments possessed wider average niche breadth and more generalist taxa, whereas above-ground tissues were dominated by narrow-niche specialists (Figure 3b). This niche differentiation pattern highlights distinct microbial adaptive strategies in different host niches. The open, resource-rich soil environment allows the survival of diverse generalist microbes with broad environmental adaptability, while the specialized, stressful endophytic niches select for host-specific specialists with strong niche adaptation (Fierer et al., 2007). Despite geographical fluctuations in the abundance of specialist and generalist taxa, the dominant status of specialist microbes was conserved across all gradients (Figures 3a,b), revealing a stable ecological assembly strategy for tobacco microbiota that prioritizes niche-specialized adaptive populations.

Integrated genomic structural and functional analyses further uncovered the adaptive mechanisms underlying specialist and generalist microbial differentiation (Figures 4, 5 and Supplementary Figure S3). Genomic structural traits, including GC content and coding density, were highly conserved between specialist and generalist MAGs across all microhabitats, with only niche-specific variations in genome size and gene length observed in bulk soil and root tissues (Figure 5). Consistently, the overall KO gene proportion and quantity showed no significant differences between the two ecological groups at both single-niche and whole-community scales, and functional PCoA ordinations exhibited substantial overlap between specialists and generalists (Figure 5 and Supplementary Figure S3). These results indicate that the core genomic architecture and basic functional repertoires are largely conserved between tobacco-associated specialist and generalist bacteria, and their ecological niche differentiation is not driven by global functional genomic divergence.

Nevertheless, niche-specific functional differentiation was detected in fine-scale KO gene profiles between specialists and generalists across microhabitats (Supplementary Figure S4). Each tobacco compartment harbored unique sets of differentially enriched functional pathways. Bulk soil microbes differed primarily in energy metabolism and xenobiotic degradation pathways, adapting to complex soil substance cycling processes (Supplementary Figure S4a). Rhizosphere and root microbes exhibited divergent amino acid metabolism, secondary metabolite biosynthesis, and drug resistance functions, which are critical for microbial rhizosphere colonization, host interaction, and stress resistance (Supplementary Figures S4b, S7c). In contrast, stem and leaf tissues only retained simple differential functional pathways related to carbohydrate, lipid, and glycan metabolism (Supplementary Figures S4d, S7e). The gradually simplified functional differentiation spectrum from soil to above-ground tissues corresponds perfectly to the intensified host filtering gradient, suggesting that host niches impose distinct functional selective pressures: complex soil environments drive microbial metabolic versatility, while specialized plant endophytic niches screen for specific host-adaptive functional traits, as described in prior studies (Russ et al., 2023).

Life-history strategy analysis further supplemented our understanding of tobacco microbiome ecological adaptation (Figures 6a–c). Y-strategists dominated both specialist and generalist microbial communities across all microhabitats and sites (Figure 6a), with specialists possessing a higher proportion of Y-strategists than generalists, while A- and S-strategists were extremely rare (Figures 6b,c). Combined with core microbiome identification, core MAGs universally adopted Y-strategy traits and exhibited obvious niche enrichment in leaf tissues (Figures 6d,e). Y-strategist microbes, characterized by rapid growth and efficient resource utilization, are highly adaptable to the dynamic and nutrient-limited endophytic environment (Cheng et al., 2024), explaining their dominant status in tobacco host niches. The highest core microbial abundance in leaf tissues and the lowest in bulk soil (Figure 6e) further indicates that host endophytic niches, particularly the phyllosphere, select for conserved core adaptive microbial lineages, while soil niches maintain more sporadic and non-core microbial populations.

## 5 Conclusion

In conclusion, this study systematically elucidates the multi-scale assembly and adaptive mechanisms of tobacco-associated bacterial microbiota. We demonstrate that tobacco microbiome assembly follows a deterministic hierarchical filtering model: geographical environments shape regional soil microbial species pools, while host microhabitat filtering drives taxonomic, diversity, functional, and life-history strategy differentiation, ultimately forming a niche-specialist-dominated microbiome with compartment-specific functional adaptation. Our findings clarify the independent and interactive effects of host and geographical factors, reveal conserved genomic functional characteristics and niche-specific adaptive divergence between specialist and generalist microbes, and highlight the dominant role of Y-strategy core microbes in host niche adaptation. These results deepen our mechanistic understanding of plant microbiome assembly rules and provide a theoretical foundation for targeted regulation of tobacco microbial communities to improve host growth and stress adaptation. In future, integrate metatranscriptomics, metaproteomics, and metabolomics with amplicon and MAG data to move beyond predictive annotation toward experimentally verified functional traits, particularly for specialist and Y-strategy core microbes.

## Data Availability

The original contributions presented in the study are publicly available. This data can be found at: https://ngdc.cncb.ac.cn/gsa/index.jsp with CRA046939.
