# Supplementary material for: Characteristics and assembly mechanisms of tobacco-associated bacteria in typical tobacco-planting regions across China
Source: Front Microbiol. 2026 Aug 25;17:1933656. doi: 10.3389/fmicb.2026.1933656 (PMC13549917; doi:10.3389/fmicb.2026.1933656)
Supplement: Supplementary file 1 [file Supplementary_file_1.docx]

import sys

import os

import collections

import argparse

A_STRATEGY_TRANSPORTERS = {

"Sulfate_Transport": [["K02048", "K23163"], ["K02046", "K02047"], ["K02045"]],

"Tungstate_Transport": [["K05772"], ["K02773", "K05773"], ["K06857"]],

"Molybdate_Transport_Wtp": [["K15495"], ["K15496"], ["K15497"]],

"nitrate_nrt_transporter": [["K15576"], ["K15577"], ["K15578"], ["K15579"]],

"bicarbonate_cmp_transporter": [["K11950"], ["K11951"], ["K11952"], ["K11953"]],

"taurine_tau_transporter": [["K15551"], ["K10831"], ["K15552"]],

"alkanesulfonate_ssu_transporter": [["K15553"], ["K15555"], ["K15554"]],

"hmp_thi_transporter": [["K15598"], ["K15599"], ["K15600"]],

"molybdate_mod_transporter": [["K02020"], ["K02018"], ["K02017"]],

"iron_III_fbp_transporter": [["K02012"], ["K02011"], ["K02010"]],

"thiamin_thi_transporter": [["K02062"], ["K02063"], ["K02064"]],

"spermidine_putrescine_abc": [["K11069"], ["K11070"], ["K11071"], ["K11072"]],

"aep_abc": [["K11081"], ["K11082"], ["K11083"], ["K11084"]],

"glycine_betaine_abc": [["K02002"], ["K02001"], ["K02000"]],

"osmoprotectant_abc": [["K05845"], ["K05846"], ["K05847"]],

"maltose_abc": [["K10108"], ["K10109"], ["K10110"], ["K10111", "K10112"]],

"galactose_oligomer_abc": [["K15770"], ["K15771"], ["K15772"], ["K10111", "K10112"]],

"raffinose_abc": [["K10117"], ["K10118"], ["K10119"], ["K10112"]],

"lactose_abc": [["K10188"], ["K10189"], ["K10190"], ["K10191"]],

"sorbitol_abc": [["K10227"], ["K10228"], ["K10229"], ["K10111", "K10112"]],

"alpha_glucoside_abc": [["K10232"], ["K10233"], ["K10234"], ["K10235", "K10112"]],

"oligogalacturonide_abc": [["K10192"], ["K10193"], ["K10194"], ["K10195"]],

"alpha_1_4_digalacturonate_abc": [["K17241"], ["K17242"], ["K17243"]],

"aldouronate_abc": [["K17318"], ["K17319"], ["K17320"]],

"trehalose_maltose_abc": [["K10236"], ["K10237"], ["K10238"], ["K10111"]],

"n_acetylglucosamine_abc": [["K10200"], ["K10201"], ["K10202"]],

"cellobiose_transporter": [["K10240"], ["K10241"], ["K10242"], ["K10112"]],

"chitobiose_das_transporter": [["K17329"], ["K17330"], ["K17331"], ["K10112"]],

"chitobiose_chi_transporter": [["K17244"], ["K17245"], ["K17246"]],

"multiple_sugar_transporter": [["K10546"], ["K10547"], ["K10548"]],

"phospholipid_mla_transporter": [["K07323"], ["K02066"], ["K02067"], ["K02065"], ["K07122"]],

"nucleoside_transporter": [["K07335"], ["K23535"], ["K23536"], ["K23537"]],

"glucose_mannose_transporter": [["K17315"], ["K17316"], ["K17317"], ["K10112"]],

"ribose_transporter": [["K10439"], ["K10440"], ["K10441"]],

"l_arabinose_transporter": [["K10537"], ["K10538"], ["K10539"]],

"galactofuranose_transporter": [["K10540"], ["K10541"], ["K10542"]],

"d_xylose_transporter": [["K10543"], ["K10544"], ["K10545"]],

"fructose_transporter": [["K10552"], ["K10553"], ["K10554"]],

"rhamnose_transporter": [["K10559"], ["K10560"], ["K10561"], ["K10562"]],

"erythritol_transporter": [["K17202"], ["K17203"], ["K17204"]],

"xylitol_transporter": [["K17205"], ["K17206"], ["K17207"]],

"myo_inositol_transporter": [["K17208", "K17213"], ["K17209", "K17214"], ["K17210", "K17215"]],

"glycerol_transporter": [["K17325"], ["K17322"], ["K17323"], ["K17324"], ["K17321"]],

"sn_glycerol_3_phosphate_transporter": [["K05813"], ["K05814"], ["K05815"], ["K05816"]],

"phosphate_transporter": [["K02040"], ["K02037"], ["K02038"], ["K02036"]],

"phosphonate_transporter": [["K02044"], ["K02042"], ["K02041"]],

"histidine_transporter": [["K10014"], ["K10015"], ["K10016"], ["K10017"]],

"glutamine_transporter": [["K10036"], ["K10037"], ["K10038"]],

"aspartate_glutamate_glutamine_transporter": [["K10039"], ["K10040"], ["K10041"]],

"arginine_transporter": [["K09996", "K09997"], ["K09998"], ["K09999"], ["K10000"]],

"glutamate_aspartate_transporter": [["K10001"], ["K10002"], ["K10003"], ["K10004"]],

"octopine_nopaline_transporter": [["K10018"], ["K10019"], ["K10020"], ["K10021"]],

"general_l_amino_acid_transporter": [["K09969"], ["K09970"], ["K09971"], ["K09972"]],

"glutamate_transporter": [["K10005"], ["K10006"], ["K10007"], ["K10008"]],

"cystine_tcyabc_transporter": [["K02424"], ["K10009"], ["K10010"]],

"cystine_tcyJKLMN_transporter": [["K16956"], ["K16957"], ["K16958"], ["K16959"], ["K16960"]],

"s_methylcysteine_transporter": [["K16961"], ["K16962"], ["K16963"]],

"arginine_ornithine_transporter": [["K10022"], ["K10023"], ["K10024"], ["K10025"]],

"arginine_lysine_histidine_transporter": [["K23059"], ["K17077"], ["K23060"]],

"multi_amino_acid_octopine_transporter": [["K23055"], ["K23056"], ["K23057"], ["K23058"]],

"branched_chain_amino_acid_transporter": [["K01999"], ["K01997"], ["K01998"], ["K01995"], ["K01996"]],

"d_methionine_transporter": [["K02073"], ["K02072"], ["K02071"]],

"urea_transporter": [["K11959"], ["K11960"], ["K11961"], ["K11962"], ["K11963"]],

"oligopeptide_transporter": [["K15580"], ["K15581"], ["K15582"], ["K15583"], ["K10823"]],

"dipeptide_heme_transporter": [["K12368"], ["K12369"], ["K12370"], ["K12371"], ["K12372"]],

"dipeptide_transporter": [["K16199"], ["K16200"], ["K16201"], ["K16202"]],

"defensin_transporter": [["K19226"], ["K19227"], ["K19228"], ["K19229"], ["K19230"]],

"nickel_nik_transporter": [["K15584"], ["K15585"], ["K15586"], ["K15587"], ["K10824"]],

"glutathione_transporter": [["K13889"], ["K13890"], ["K13891"], ["K13892"]],

"microcin_c_transporter": [["K13893"], ["K13894"], ["K13895"], ["K13896"]],

"fe3_dicitrate_transporter": [["K23181"], ["K23183"], ["K23182"], ["K23184"]],

"fe_siderophore_transporter": [["K23185", "K25286"], ["K23186"], ["K23187"], ["K23188"]],

"fe3_hydroxamate_transporter": [["K23227"], ["K23228"], ["K10829"]],

"vitamin_b12_transporter": [["K06858"], ["K06073"], ["K06074"]],

"manganese_transporter": [["K19975"], ["K19976"], ["K19973"]],

"zinc_transporter": [["K09815"], ["K09816"], ["K09817"]],

"iron3_manganese_sit_transporter": [["K11604"], ["K11605"], ["K11606"], ["K11607"]],

"zinc_manganese_iron3_tro_transporter": [["K11707"], ["K11708"], ["K11709"], ["K11710"]],

"cobalt_transporter": [["K02009"], ["K02007"], ["K02008"], ["K02006"]],

"nickel_cbi_transporter": [["K10094"], ["K02009"], ["K16915"], ["K02007"], ["K02008"], ["K02006"]],

"biotin_bio_transporter": [["K03523"], ["K16783"], ["K16784"]],

"biotin_ecf_transporter": [["K03523"], ["K16785"], ["K16786"], ["K16787"]],

"autoinducer_2_transporter": [["K10555"], ["K10556"], ["K10557"], ["K10558"]]

}

PTS_ENGINE = ["K08483"]

PTS_HPR_GENERAL = ["K02784"]

PTS_HPR_FRUCTOSE = ["K02768"]

PTS_PLUGINS = {

"Glucose_PTS": [["K02777"], ["K02779"]],

"Fructose_PTS": [["K02769"], ["K02770"]]

}

DHBA_CORE = [["K02361", "K02552", "K01851"], ["K01252"], ["K00216"]]

SIDEROPHORE_BIOSYNTHESIS = {

"Enterobactin": DHBA_CORE + [["K02363"], ["K02362"], ["K02364"]],

"Vibriobactin": DHBA_CORE + [["K02363"], ["K04778"], ["K12237"]],

"Bacillibactin": DHBA_CORE + [["K02363"], ["K04780"]],

"Myxochelin": DHBA_CORE + [["K02363"], ["K15653"], ["K15681"]],

"Pyochelin": [["K01851"], ["K04782"], ["K12238"], ["K12239"], ["K12240"], ["K12241"]],

"Yersiniabactin": [["K04781"], ["K04783"], ["K04784"], ["K04785"], ["K04786"]],

"Mycobactin": [["K04781"], ["K04787"], ["K04788"], ["K04789"], ["K04790"], ["K04791"], ["K04792"], ["K04793"]]

}

FLAGELLAR_CORE = [

["K02409"], ["K02410"], ["K02400"], ["K02412"], ["K02390"], ["K02406"],

["K02556", "K10564"], ["K02557", "K10565"]

]

CHEMOTAXIS_CORE = [

["K03406", "K05874", "K05875", "K05876", "K05877", "K03776"],

["K03408", "K03415"], ["K03407"], ["K03413"], ["K00575"], ["K03412", "K13924"]

]

RNA_DEGRADOSOME = [["K08300", "K18682", "K12574"], ["K00962", "K12573"], ["K03732", "K11927", "K05592"]]

SRNA_HUB = [["K03666"], ["K08300", "K18682", "K12574"]]

T1SS = [["K12340"], ["K11003"], ["K11004"]]

T2SS = [["K02453"], ["K02455"], ["K02454"], ["K02456"]]

def read_ko_profile(filepath):

ko_set = set()

try:

with open(filepath, 'r') as f:

for line in f:

parts = line.strip().split('\t')

if len(parts) >= 2:

ko = parts[0]

try:

count = float(parts[1])

if count > 0:

ko_set.add(ko)

except ValueError:

continue

except Exception as e:

sys.stderr.write(f"Error reading file {filepath}: {e}\n")

return ko_set

def check_module_completeness(module_list, ko_set, threshold):

""" 计算基于模块嵌套列表的完整度 (存在数/总数 >= 阈值) """

if not module_list:

return False

total_blocks = len(module_list)

present_blocks = sum(1 for sub_group in module_list if any(ko in ko_set for ko in sub_group))

return (present_blocks / total_blocks) >= (threshold / 100.0)

def evaluate_a_strategy(ko_set, threshold):

report = collections.defaultdict(list)

a_score = 0.0

\# 1. ABC (+1.0)

for name, module in A_STRATEGY_TRANSPORTERS.items():

if check_module_completeness(module, ko_set, threshold):

a_score += 1.0

report['ABC_Transporters'].append((name, 1.0))

\# 2. PTS (+2.0)

has_pts_engine = any(ko in ko_set for ko in PTS_ENGINE)

if has_pts_engine:

if any(ko in ko_set for ko in PTS_HPR_GENERAL) and check_module_completeness(PTS_PLUGINS["Glucose_PTS"], ko_set, threshold):

a_score += 2.0

report['PTS_Systems'].append(("Glucose_PTS", 2.0))

if any(ko in ko_set for ko in PTS_HPR_FRUCTOSE) and check_module_completeness(PTS_PLUGINS["Fructose_PTS"], ko_set, threshold):

a_score += 2.0

report['PTS_Systems'].append(("Fructose_PTS", 2.0))

\# 3. 铁载体 (+3.0)

for name, module in SIDEROPHORE_BIOSYNTHESIS.items():

if check_module_completeness(module, ko_set, threshold):

a_score += 3.0

report['Siderophore_Biosynthesis'].append((name, 3.0))

\# 4. 运动与感知

has_flagella = check_module_completeness(FLAGELLAR_CORE, ko_set, threshold)

has_chemotaxis = check_module_completeness(CHEMOTAXIS_CORE, ko_set, threshold)

if has_flagella:

a_score += 3.0

report['Motility_and_Sensing'].append(("Flagellar_Assembly", 3.0))

if has_chemotaxis:

if has_flagella:

a_score += 5.0 # (2.0基础 + 3.0协同增益)

report['Motility_and_Sensing'].append(("Chemotaxis (Synergistic Bonus)", 5.0))

else:

a_score += 1.0

report['Motility_and_Sensing'].append(("Chemotaxis (Orphaned)", 1.0))

if check_module_completeness(RNA_DEGRADOSOME, ko_set, threshold):

a_score += 2.0

report['Responsiveness'].append(("RNA_Degradosome", 2.0))

if check_module_completeness(SRNA_HUB, ko_set, threshold):

a_score += 2.0

report['Responsiveness'].append(("sRNA_Regulatory_Hub", 2.0))

if check_module_completeness(T1SS, ko_set, threshold):

a_score += 1.0

report['Secretion_Acquisition'].append(("T1SS", 1.0))

if check_module_completeness(T2SS, ko_set, threshold):

a_score += 3.0

report['Secretion_Acquisition'].append(("T2SS", 3.0))

return a_score, report

if __name__ == "__main__":

parser = argparse.ArgumentParser(description="Calculate A-score for MAG(s) based on KO profile.")

parser.add_argument("-i", "--input", required=True, nargs='+', help="Path to the MAG KO TSV file(s). Supports wildcards.", type=str)

parser.add_argument("-t", "--threshold", type=float, default=70.0, help="Completeness threshold percentage (Default: 70.0)")

parser.add_argument("-o", "--output", help="Optional: Path to save detailed scoring report with sub-scores", type=str)

args = parser.parse_args()

if args.output:

with open(args.output, 'w', encoding='utf-8') as f:

f.write(f"A-Score Detailed Report (Threshold: {args.threshold}%)\n")

f.write("=" * 50 + "\n\n")

for filepath in args.input:

if os.path.isdir(filepath):

continue

if not os.path.exists(filepath):

sys.stderr.write(f"Warning: File '{filepath}' not found, skipping.\n")

continue

mag_id = os.path.splitext(os.path.basename(filepath))[0]

mag_kos = read_ko_profile(filepath)

total_a_score, details = evaluate_a_strategy(mag_kos, args.threshold)

print(f"{mag_id}\t{total_a_score:.1f}")

if args.output:

with open(args.output, 'a', encoding='utf-8') as f:

f.write(f"MAG ID: {mag_id}\n")

f.write(f"Total A-Score: {total_a_score:.1f}\n")

f.write("-" * 40 + "\n")

if not details:

f.write("No A-Strategy modules assembled.\n")

else:

for category, module_scores in details.items():

category_total = sum(score for name, score in module_scores)

f.write(f"[{category}] - Sub-total: {category_total:.1f}\n")

for mod_name, mod_score in module_scores:

f.write(f" - {mod_name} (+{mod_score:.1f})\n")

f.write("\n" + "=" * 50 + "\n\n")
