# Supplementary material for: Characteristics and assembly mechanisms of tobacco-associated bacteria in typical tobacco-planting regions across China
Source: Front Microbiol. 2026 Aug 25;17:1933656. doi: 10.3389/fmicb.2026.1933656 (PMC13549917; doi:10.3389/fmicb.2026.1933656)
Supplement: Supplementary file 2 [file Supplementary_file_2.docx]

import sys

import os

import re

import collections

import argparse

S_STRATEGY_PATHWAYS = {

"Homologous_Recombination": [

["K03553"], ["K03582", "K03584", "K03629"], ["K01159", "K03655"]

],

"Base_Excision_Repair": [

["K03660", "K10773", "K03648", "K03575", "K03652", "K10563", "K05522", "K01247", "K13529", "K01246", "K03649", "K21929"],

["K01142", "K01151"]

],

"Nucleotide_Excision_Repair": [

["K03701"], ["K03702"], ["K03703"]

],

"LPS_Biosynthesis": [

["K02535", "K16363"], ["K00748"], ["K02527"]

],

"Peptidoglycan_Biosynthesis": [

["K00790", "K01928", "K05362", "K01929", "K15792", "K01798"],

["K01000"], ["K02563"],

["K05366", "K05365", "K03693", "K03587", "K05515", "K03814"]

],

"Mismatch_Repair": [

["K03555"], ["K03572"]

],

"Proteasome": [

['K03432'], ['K03433'], ['K13527', 'K03420']

]

}

Ascorbate_Aldarate = {

"Ascorbate_utilization": [["K03476"], ["K03078", "K03079"]],

"Aldarate_utilization": [["K01706", "K01708", "K01707"], ["K01630"]]

}

Porphyrin_Metabolism = {

"Heme_biosynthesis": [["K01698"], ["K01749"], ["K01599"], ["K01772"]],

"Cobalamin_biosynthesis": [

["K02302", "K02303", "K13542"],

["K02190", "K03795", "K02230"],

["K02233", "K09882"]

]

}

\# =====================================================================

\# 2. Evaluation

\# =====================================================================

def read_ko_profile(filepath):

"""

智能解析器：兼容 KEGG Mapper 列表、GhostKOALA、以及带丰度的 TSV。

如果是丰度矩阵，会自动过滤掉丰度 <= 0 的行。

"""

ko_set = set()

try:

with open(filepath, 'r', encoding='utf-8') as f:

for line in f:

parts = line.strip().split('\t')

if len(parts) >= 2 and parts[0].startswith('K') and len(parts[0]) == 6:

try:

if float(parts[1]) <= 0:

continue

except ValueError:

pass # 如果第二列不是数字（比如 GhostKOALA 格式），继续往下走

kos = re.findall(r'K\d{5}', line)

ko_set.update(kos)

except Exception as e:

sys.exit(f"Error reading file {filepath}: {e}")

return ko_set

def check_module_completeness(module_list, ko_set, threshold):

if not module_list:

return False

total_blocks = len(module_list)

present_blocks = 0

for sub_group in module_list:

if any(ko in ko_set for ko in sub_group):

present_blocks += 1

completeness_ratio = present_blocks / total_blocks

return completeness_ratio >= (threshold / 100.0)

def calculate_s_score(ko_set, threshold):

report = collections.defaultdict(list)

active_pathways = 0

for name, mvc in S_STRATEGY_PATHWAYS.items():

if check_module_completeness(mvc, ko_set, threshold):

active_pathways += 1

report["Active_Pathways"].append(name)

else:

report["Inactive_Pathways"].append(name)

asc = check_module_completeness(Ascorbate_Aldarate["Ascorbate_utilization"], ko_set, threshold)

ald = check_module_completeness(Ascorbate_Aldarate["Aldarate_utilization"], ko_set, threshold)

if asc or ald:

active_pathways += 1

report["Active_Pathways"].append("Ascorbate_Aldarate_Metabolism")

else:

report["Inactive_Pathways"].append("Ascorbate_Aldarate_Metabolism")

heme = check_module_completeness(Porphyrin_Metabolism["Heme_biosynthesis"], ko_set, threshold)

cob = check_module_completeness(Porphyrin_Metabolism["Cobalamin_biosynthesis"], ko_set, threshold)

if heme or cob:

active_pathways += 1

report["Active_Pathways"].append("Porphyrin_Metabolism")

else:

report["Inactive_Pathways"].append("Porphyrin_Metabolism")

total_pathways = len(S_STRATEGY_PATHWAYS) + 2

s_score_normalized = active_pathways / total_pathways

return s_score_normalized, active_pathways, total_pathways, report

if __name__ == "__main__":

parser = argparse.ArgumentParser(description="Calculate S-score for a single MAG based on KO profile.")

parser.add_argument("-i", "--input", required=True, type=str, help="Input file containing KOs (TSV or List)")

parser.add_argument("-t", "--threshold", type=float, default=70.0, help="Completeness threshold percentage (Default: 70.0)")

parser.add_argument("-o", "--output", type=str, help="Optional: Path to save detailed scoring report")

args = parser.parse_args()

if not os.path.exists(args.input):

sys.exit(f"Error: Input file '{args.input}' not found.")

mag_id = os.path.splitext(os.path.basename(args.input))[0]

ko_set = read_ko_profile(args.input)

if not ko_set:

sys.stderr.write(f"Warning: No valid KOs found in '{args.input}'.\n")

s_score, active, total, details = calculate_s_score(ko_set, args.threshold)

print(f"{mag_id}\t{s_score:.4f}")

if args.output:

try:

with open(args.output, 'w', encoding='utf-8') as f_out:

f_out.write(f"MAG ID: {mag_id}\n")

f_out.write(f"Total KOs Detected: {len(ko_set)}\n")

f_out.write(f"Completeness Threshold: {args.threshold}%\n")

f_out.write(f"Active S-Pathways: {active} / {total}\n")

f_out.write(f"Final S-Score: {s_score:.4f}\n")

f_out.write("-" * 40 + "\n")

f_out.write("[ACTIVE PATHWAYS]:\n")

for p in details.get("Active_Pathways", []):

f_out.write(f" + {p}\n")

f_out.write("\n[INACTIVE PATHWAYS]:\n")

for p in details.get("Inactive_Pathways", []):

f_out.write(f" - {p}\n")

except Exception as e:

sys.stderr.write(f"Error writing to output report {args.output}: {e}\n")
