# Supplementary material for: Characteristics and assembly mechanisms of tobacco-associated bacteria in typical tobacco-planting regions across China
Source: Front Microbiol. 2026 Aug 25;17:1933656. doi: 10.3389/fmicb.2026.1933656 (PMC13549917; doi:10.3389/fmicb.2026.1933656)
Supplement: Supplementary file 3 [file Supplementary_file_3.docx]

import argparse

import csv

import sys

import os

Y_STRATEGY_MODULES = {

\# 碳水化合物与核心能量代谢

"M00001": [["K16370", "K00850", "K00918"], ["K00873", "K12406"]], # Glycolysis (Embden-Meyerhof pathway), glucose => pyruvate

"M00003": [["K03841", "K01622"], ["K01610"]], # Gluconeogenesis, oxaloacetate => fructose-6P

"M00004": [["K00036"], ["K00033"]], # Pentose phosphate pathway (Pentose phosphate cycle)

"M00005": [[]], # PRPP biosynthesis, ribose-5P => PRPP (单基因)

"M00006": [["K00036", "K19243", "K13937"], ["K00033"]], # Pentose phosphate pathway, oxidative phase, glucose-6P => ribulose-5P

"M00007": [["K00615"], ["K00616"]], # Pentose phosphate pathway, non-oxidative phase, fructose-6P => ribose-5P

"M00009": [["K01647", "K05942", "K01659"], ["K00031", "K00030"]], # TCA cycle

"M00010": [[]], # Citrate cycle, first carbon oxidation, oxaloacetate => 2-oxoglutarate

"M00011": [["K00239"], ["K00240"]], # Citrate cycle, second carbon oxidation, 2-oxoglutarate => oxaloacetate

"M00307": [[]], # Pyruvate oxidation, pyruvate => acetyl-CoA

\# 脂肪酸代谢

"M00082": [["K01962", "K02160", "K01961", "K01963", "K11262", "K11263"], ["K00665", "K11533", "K00648", "K18473"]], # Fatty acid biosynthesis, initiation

"M00083": [["K11533", "K00647", "K09458"], ["K11533", "K00208", "K02371", "K10780", "K00209"]], # Fatty acid biosynthesis, elongation

"M00086": [["K01897", "K15013", "K28161"]], # beta-Oxidation, acyl-CoA synthesis

\# 碳固定

"M00165": [["K01601"], ["K00855", "K28462"]], # Reductive pentose phosphate cycle (Calvin cycle)

"M00173": [["K15230", "K15231", "K15234"]], # Reductive citrate cycle (Arnon-Buchanan cycle)

"M00377": [["K00198"], ["K14138", "K00197", "K00194"]], # Reductive acetyl-CoA pathway (Wood-Ljungdahl pathway)

"M00376": [["K14468", "K14469"]], # 3-Hydroxypropionate bi-cycle

\# 氨基酸合成

"M00019": [["K00053", "K01687"]], # Valine/isoleucine biosynthesis, pyruvate => valine / 2-oxobutanoate => isoleucine

"M00432": [["K01649"], ["K00052", "K01703", "K01704"]], # Leucine biosynthesis, 2-oxoisovalerate => 2-oxoisocaproate

"M00570": [["K01754", "K17989"]], # Isoleucine biosynthesis, threonine => 2-oxobutanoate => isoleucine

"M00535": [["K09011"]], # Isoleucine biosynthesis, pyruvate => 2-oxobutanoate

\# 芳香族及其他复杂合成代谢

"M00016": [["K00674", "K01439"]], # Lysine biosynthesis, succinyl-DAP pathway, aspartate => lysine

"M00525": [["K05822", "K05823"]], # Lysine biosynthesis, acetyl-DAP pathway, aspartate => lysine

"M00526": [["K03340"]], # Lysine biosynthesis, DAP dehydrogenase pathway, aspartate => lysine

"M00527": [["K10206"]], # Lysine biosynthesis, DAP aminotransferase pathway, aspartate => lysine

"M00022": [["K01735", "K00891", "K00800", "K01736"]], # Shikimate pathway, phosphoenolpyruvate + erythrose-4P => chorismate

"M00023": [["K00766", "K01817", "K01609", "K01694", "K01695", "K01696"]], # Tryptophan biosynthesis, chorismate => tryptophan

"M00024": [["K01713"], ["K04518"], ["K05359"]], # Phenylalanine biosynthesis, chorismate => phenylpyruvate => phenylalanine

"M00025": [["K04517", "K00211", "K14187"]], # Tyrosine biosynthesis, chorismate => HPP => tyrosine/Tyrosine biosynthesis, chorismate => arogenate => tyrosine

"M00040": [["K00220", "K24018", "K15227"]], # 用户注: M00025/M00040 同门控

\# 辅酶及其他合成

"M00912": [["K00453", "K00463", "K00486", "K01556", "K00452"]], # NAD biosynthesis, tryptophan => quinolinate => NAD

"M00038": [["K03392", "K10217", "K23234"]], # Tryptophan metabolism, tryptophan => kynurenine => 2-aminomuconate

"M00126": [["K01633", "K13939", "K13940", "K13941", "K00950", "K00796", "K11754", "K20457"]], # Tetrahydrofolate biosynthesis, GTP => THF

"M00140": [["K01491", "K00300", "K01500"]], # C1-unit interconversion, prokaryotes

"M00621": [["K00605"], ["K00281", "K00282", "K00283"]], # Glycine cleavage system

"M00555": [["K17755", "K00108", "K11440", "K00499"], ["K17755", "K00130", "K14085"]], # Betaine biosynthesis, choline => betaine

"M00035": [["K00789"], ["K01697", "K10150"]], # Methionine degradation

"M00338": [["K01697", "K10150"], ["K01758"]] # Cysteine biosynthesis, homocysteine + serine => cysteine

}

def load_module_completeness(file_path):

"""读取 Input 1: MAG的Module完整度"""

modules = {}

with open(file_path, 'r', encoding='utf-8') as f:

\# 兼容以制表符或逗号分隔的文件

reader = csv.reader(f, delimiter='\t')

for row in reader:

if len(row) < 2:

continue

mod_id = row[0].strip()

\# 过滤非Module编号的表头

if not mod_id.startswith('M') or not mod_id[1:].isdigit():

continue

try:

\# 兼容 0.85 或 85(%) 格式的完整度，统一转为浮点数

completeness = float(row[1].strip().replace('%', ''))

modules[mod_id] = completeness

except ValueError:

continue

return modules

def load_ko_counts(file_path):

"""读取 Input 2: MAG的KEGG KO 计数"""

present_kos = set()

with open(file_path, 'r', encoding='utf-8') as f:

reader = csv.reader(f, delimiter='\t')

for row in reader:

if len(row) < 2:

continue

ko_id = row[0].strip()

if not ko_id.startswith('K') or not ko_id[1:].isdigit():

continue

try:

count = int(row[1].strip())

if count >= 1:

present_kos.add(ko_id)

except ValueError:

continue

return present_kos

def calculate_y_score(module_completeness, present_kos, threshold):

"""计算单个 MAG 的 Y-Score 详情"""

results = []

total_y_score = 0.0

for mod_id, gating_logic in Y_STRATEGY_MODULES.items():

raw_comp = module_completeness.get(mod_id, 0.0)

if raw_comp < threshold:

results.append({

"Module": mod_id,

"Raw_Completeness": raw_comp,

"Gating_Passed": "False (Module Missing)",

"Missing_KOs": "-",

"Effective_Score": 0.0

})

continue

if raw_comp == 0:

results.append({

"Module": mod_id,

"Raw_Completeness": 0.0,

"Gating_Passed": "False (Module Missing)",

"Missing_KOs": "-",

"Effective_Score": 0.0

})

continue

gating_passed = True

missing_gates = []

for required_group in gating_logic:

if not required_group:

continue

if not any(ko in present_kos for ko in required_group):

gating_passed = False

missing_gates.append("/".join(required_group))

if gating_passed:

effective_score = raw_comp

status = "True"

missing_str = "None"

else:

effective_score = 0.0

status = "False (Toll-gate Blocked)"

missing_str = ", ".join(missing_gates)

total_y_score += effective_score

results.append({

"Module": mod_id,

"Raw_Completeness": raw_comp,

"Gating_Passed": status,

"Missing_KOs": missing_str,

"Effective_Score": effective_score

})

return total_y_score, results

def main():

parser = argparse.ArgumentParser(description="Calculate MAG Y-Score based on Module Completeness and KO counts.")

parser.add_argument("-m", "--modules", required=True, help="Input 1: Module completeness file (TSV/CSV)")

parser.add_argument("-k", "--kos", required=True, help="Input 2: KO counts file (TSV/CSV)")

parser.add_argument("-o", "--output", required=True, help="Output detailed TSV file path")

parser.add_argument("-t", "--threshold", type=float, default=70.0, help="Completeness threshold (e.g. 70 for 70% or 0.7 depending on your input format). Default is 70.0.")

args = parser.parse_args()

if not os.path.exists(args.modules):

sys.exit(f"Error: Module file '{args.modules}' not found.")

if not os.path.exists(args.kos):

sys.exit(f"Error: KO file '{args.kos}' not found.")

mag_id = os.path.splitext(os.path.basename(args.modules))[0]

print(f"Loading Module data from: {args.modules}")

mod_comp = load_module_completeness(args.modules)

print(f"Loading KO data from: {args.kos}")

present_kos = load_ko_counts(args.kos)

print("Evaluating Y-Strategy Toll-gates...")

total_score, detailed_results = calculate_y_score(mod_comp, present_kos, args.threshold)

with open(args.output, 'w', encoding='utf-8', newline='') as f:

writer = csv.writer(f, delimiter='\t')

writer.writerow(["Module", "Raw_Completeness", "Gating_Passed", "Missing_Gating_KOs", "Effective_Y_Score"])

for res in detailed_results:

writer.writerow([

res["Module"],

res["Raw_Completeness"],

res["Gating_Passed"],

res["Missing_KOs"],

res["Effective_Score"]

])

#print("-" * 50)

#print(f"Analysis Complete!")

#print(f"Total Y-Strategy Modules Evaluated: {len(Y_STRATEGY_MODULES)}")

#print(f"Total Effective Y-Score for this MAG: {total_score:.2f}")

print(f"{mag_id}\t{total_score:.2f}")

#print(f"Detailed output saved to: {args.output}")

#print("-" * 50)

if __name__ == "__main__":

main()
