# Supplementary material for: Characteristics and assembly mechanisms of tobacco-associated bacteria in typical tobacco-planting regions across China
Source: Front Microbiol. 2026 Aug 25;17:1933656. doi: 10.3389/fmicb.2026.1933656 (PMC13549917; doi:10.3389/fmicb.2026.1933656)
Supplement: Supplementary file 4 [file Supplementary_file_4.docx]

import pandas as pd

from sklearn.preprocessing import MinMaxScaler

import argparse

import sys

import os

def main():

\# ==========================================

\# 1. 设置命令行参数解析 (argparse)

\# ==========================================

parser = argparse.ArgumentParser(description="Normalize Y-A-S scores and determine the dominant strategy for MAGs.")

\# 必须参数

parser.add_argument("-i", "--input", required=True, help="Input CSV file containing the scores")

\# 可选参数：输出文件名

parser.add_argument("-o", "--output", default="mag_strategies_classified.csv", help="Output CSV file name (default: mag_strategies_classified.csv)")

parser.add_argument("--sep", default="\t", help="Delimiter used in the files (default: tab)")

\# 可选参数：定义您的 CSV 表头叫什么（防止您的表头不是默认的 Y_score, A_score...）

parser.add_argument("--id_col", default="MAG_ID", help="Column name for MAG ID (default: MAG_ID)")

parser.add_argument("--y_col", default="Y_score", help="Column name for Y score (default: Y_score)")

parser.add_argument("--a_col", default="A_score", help="Column name for A score (default: A_score)")

parser.add_argument("--s_col", default="S_score", help="Column name for S score (default: S_score)")

args = parser.parse_args()

delimiter = '\t' if args.sep == '\\t' or args.sep == '\t' else args.sep

if not os.path.exists(args.input):

sys.exit(f"Error: Input file '{args.input}' not found.")

\# ==========================================

\# 2. 读取数据与列检查

\# ==========================================

try:

df = pd.read_csv(args.input, sep=delimiter)

except Exception as e:

sys.exit(f"Error reading the input file: {e}")

required_columns = [args.id_col, args.y_col, args.a_col, args.s_col]

for col in required_columns:

if col not in df.columns:

sys.exit(f"Error: Column '{col}' not found in the CSV. Please check your headers or use --y_col, --a_col, etc., to specify them.")

\# ==========================================

\# 3. Min-Max

\# ==========================================

scaler = MinMaxScaler()

df[['Y_norm', 'A_norm', 'S_norm']] = scaler.fit_transform(df[[args.y_col, args.a_col, args.s_col]])

\# ==========================================

\# 4. Percentage

\# ==========================================

df['Total_Investment'] = df['Y_norm'] + df['A_norm'] + df['S_norm']

df['Total_Investment'] = df['Total_Investment'].replace(0, 1)

df['Y_pct'] = df['Y_norm'] / df['Total_Investment']

df['A_pct'] = df['A_norm'] / df['Total_Investment']

df['S_pct'] = df['S_norm'] / df['Total_Investment']

def determine_strategy(row):

\# 找到比例最大的那个列的名称

max_col = row[['Y_pct', 'A_pct', 'S_pct']].idxmax()

if max_col == 'Y_pct':

return 'Y-Strategist'

elif max_col == 'A_pct':

return 'A-Strategist'

else:

return 'S-Strategist'

df['Dominant_Strategy'] = df.apply(determine_strategy, axis=1)

df.to_csv(args.output, index=False)

print(f"\n[+] Analysis complete! Results saved to: {args.output}")

print("\nPreview of classifications:")

preview_cols = [args.id_col, 'Dominant_Strategy', 'Y_pct', 'A_pct', 'S_pct']

print(df[preview_cols].head())

if __name__ == "__main__":

main()
